# Elecsys CSF AD immunoassays: Sample stability for a new pre‐analytical protocol for fresh CSF

**DOI:** 10.1002/alz.70797

**Published:** 2025-10-17

**Authors:** Ekaterina Manuilova, Isabelle Schrurs, Sandra Rutz, Silja McIlwrick, Oliver Goldhardt, Patrick Sommer, Timo Grimmer

**Affiliations:** ^1^ Roche Diagnostics GmbH Penzberg Germany; ^2^ Roche Diagnostics International Ltd Rotkreuz Switzerland; ^3^ Technical University of Munich (TUM) School of Medicine and Health TUM University Hospital Munich Germany

**Keywords:** Aβ42, Alzheimer's disease, biomarkers, Elecsys CSF immunoassay, fresh CSF, pre‐analytical handling, p‐tau, sample stability, storage conditions, t‐tau

## Abstract

**INTRODUCTION:**

Amyloid beta 1–42 (Aβ42), tau phosphorylated at threonine‐181 (p‐tau181), and total tau (t‐tau) are cerebrospinal fluid (CSF) biomarkers for Alzheimer's disease diagnosis.

**METHODS:**

We prospectively examined storage time, temperature, and freeze/thaw effects on Aβ42, p‐tau181, and t‐tau stability in fresh CSF samples using Elecsys^®^ CSF immunoassays.

**RESULTS:**

All three biomarkers were stable at 2–8°C for ≤15 days, and −25°C to −15°C for ≤8 weeks, and after one freeze/thaw cycle. p‐Tau181 and t‐tau were also stable at 15–25°C for ≤8 days and at −25°C to −15°C for 12–15 weeks. Aβ42 recovery declined slightly after storage at 15–25°C for ≤8 days and −25°C to −15°C for 12–15 weeks, and one freeze/thaw cycle.

**DISCUSSION:**

For optimal immunoassay performance, it is recommended to store CSF samples at 15–25°C for ≤5 days, 2–8°C for ≤15 days, and −25°C to −15°C for ≤8 weeks.

**Highlights:**

- Cerebrospinal fluid (CSF) biomarkers aid in Alzheimer's disease diagnosis.
- Fresh CSF stored at 15–25°C for ≤5 days is optimal for Elecsys CSF immunoassays.
- Fresh CSF stored at 2–8°C for ≤15 days is optimal for Elecsys CSF immunoassays.
- Fresh CSF stored at −25°C to −15°C for ≤8 weeks is optimal for Elecsys CSF immunoassays.

## 1 BACKGROUND

Alzheimer's disease (AD) is a progressive, irreversible, neurodegenerative brain disease and the leading cause of dementia, accounting for 60–80% of all cases.^1^, ^2^ AD is characterized by the accumulation of amyloid beta (Aβ) plaques and the hyperphosphorylation of tau proteins, which leads to the formation of neurofibrillary tangles in the brain.^3^ Several studies have reported that the levels of soluble oligomeric forms of Aβ and tau are altered in the diseased brain and can be detected in cerebrospinal fluid (CSF), showing high correlation with cognitive decline.^4^ Therefore, Aβ and tau have been identified as biomarkers for AD diagnosis using CSF samples.^5^

**RESEARCH IN CONTEXT**

1. **Systematic review**: Studies evaluating the effect of pre‐analytical factors on the stability of amyloid beta 1–42 (Aβ42), tau phosphorylated at a threonine residue at position 181 (p‐tau181), and total tau (t‐tau) in cerebrospinal fluid (CSF) were reviewed. The optimal storage conditions for fresh CSF samples measured using the Elecsys^®^ CSF immunoassays and handled with the manufacturer‐recommended protocol have not yet been identified.
2. **Interpretation**: p‐Tau181 and t‐tau were stable under all examined storage conditions; Aβ42 recovery declined slightly after storage at 15–25°C for 8 days and at −25°C to −15°C for 12–15 weeks. It is recommended to store fresh CSF samples at 15–25°C for ≤5 days, at 2–8°C for ≤15 days, and at −25°C to −15°C for ≤8 weeks.
3. **Future directions**: Additional studies need to explore the effect of temperatures < −25°C and storage >15 weeks on the stability of Aβ42, p‐tau181, and t‐tau in CSF.

Key CSF biomarkers for AD include amyloid beta 1–42 (Aβ42), which is inversely correlated with cerebral amyloid plaque burden, as well as tau phosphorylated at a threonine residue at position 181 (p‐tau181) and total tau (t‐tau), which are biomarkers for cerebral tangle formation of tau and neuronal degeneration, respectively.^3^, ^5^ Specifically, Aβ42, p‐tau181, and t‐tau are robust biomarkers for detecting amyloid pathology, supporting the timely and accurate diagnosis of AD.^6^, ^7^, ^8^, ^9^, ^10^, ^11^ In addition, recent studies have shown that the highest diagnostic power of the three biomarkers for early‐stage AD is achieved through the combination of their measurements rather than their individual levels.^12^, ^13^, ^14^, ^15^

Until recently, approved treatment options for AD included molecules that provided limited symptomatic relief and did not impact progression.^16^, ^17^ More recent advances include disease‐modifying treatments that can delay AD progression, such as lecanemab and donanemab, which are known for a remarkably high Aβ clearance.^18^, ^19^, ^20^ These developments indicate an increasing need for accurate diagnostic tests to allow earlier diagnosis of AD, giving patients time to plan for the future and access treatments that prevent or delay cognitive decline.^21^

Elecsys^®^ β‐Amyloid (1–42) CSF II, Elecsys Phospho‐Tau (181P) CSF, and Elecsys Total‐Tau CSF immunoassays (Roche Diagnostics International Ltd, Rotkreuz, Switzerland) are electrochemiluminescence assays that employ a quantitative sandwich principle and are approved for clinical use in Europe and the United States.^22^, ^23^ These immunoassays have been developed for the measurement of Aβ42, p‐tau181, and t‐tau, respectively, as an aid to detect amyloid pathology and support the timely diagnosis of AD,^22^, ^23^, ^24^ demonstrating excellent analytical performance with high precision, good lot‐to‐lot comparability, and low variability between and within laboratories.^24^ In addition, these immunoassays have shown high concordance with amyloid classification based on visual positron emission tomography (PET) read across different AD populations and pre‐analytical protocols.^25^, ^26^, ^27^, ^28^ These findings support the use of CSF biomarkers in the early identification of amyloid positivity in individuals with AD.

Although these CSF biomarkers have demonstrated clinical utility in the diagnosis of AD, pre‐analytical handling may influence biomarker levels and consequently the diagnostic accuracy of biomarker‐based tests.^26^ Among the three key CSF biomarkers for AD, Aβ42 is known to be particularly sensitive to pre‐analytical handling and storage conditions (e.g., storage time and temperature, freeze/thaw cycles, and collection tube type) due to its tendency to adhere to and aggregate on various surfaces.^26^, ^29^, ^30^, ^31^ Sample stability results may also be immunoassay‐specific and depend on the pre‐analytical protocol of CSF sample collection.^32^

Recently, a robust, easy‐to‐use, standardized, pre‐analytical protocol was developed for collecting fresh CSF samples and measuring AD biomarkers in clinical practice. This routine‐use protocol ensures robust measurements by reducing handling steps to a minimum, and using sterile low‐bind tubes and an optimal fill volume.^33^ In this prospective study, we used the Elecsys CSF immunoassays to investigate the effect of storage time, storage temperature, and freeze/thaw cycles on the stability of the Aβ42, p‐tau181, and t‐tau biomarkers in freshly collected CSF samples, according to the procedure recommended in the manufacturer‐ protocol and the respective immunoassay method sheets. The aim of this study was to identify the optimal storage conditions for CSF samples, collected with the established pre‐analytical protocol for fresh CSF and measured using the Elecsys CSF immunoassays, to support the use of these immunoassays in clinical practice.

## 2 METHODS

### 2.1 Study design

Fresh CSF samples were collected from subjects being evaluated for AD, including any stage of cognitive impairment (subjective or objective), who received a spinal tap (lumbar puncture) as part of their clinical routine. This subject group represented the intended‐use population of the Elecsys CSF immunoassays and constituted the primary population of this study. In cases where obtaining desired biomarker level ranges was challenging, other sample sources were considered, including samples from subjects with no cognitive complaints (normal CSF biomarker levels), who received spinal anesthesia for other reasons and who were willing to provide CSF sample material for study purposes, and samples from subjects under evaluation for, or with a diagnosis of, normal pressure hydrocephalus, who received a spinal tap (lumbar puncture) as part of their clinical routine. However, no subject was recruited in this study following these procedures, that is, spinal tap for the evaluation of normal pressure hydrocephalus or spinal anesthesia. Additional selection criteria included age at sample collection ≥50 years, signed informed consent, available Mini‐Mental State Examination (MMSE) score, and CSF collected at the study site following the routine collection procedure. Each subject was assigned to one of three CSF sample stability experiments: short‐term at room temperature (15–25°C), short‐term at cooled temperature (2–8°C), or mid‐term at −25°C to −15°C.[Fig alz70797-fig-0001]

### 2.2 Sample collection and measurement

**FIGURE 1 alz70797-fig-0001:**
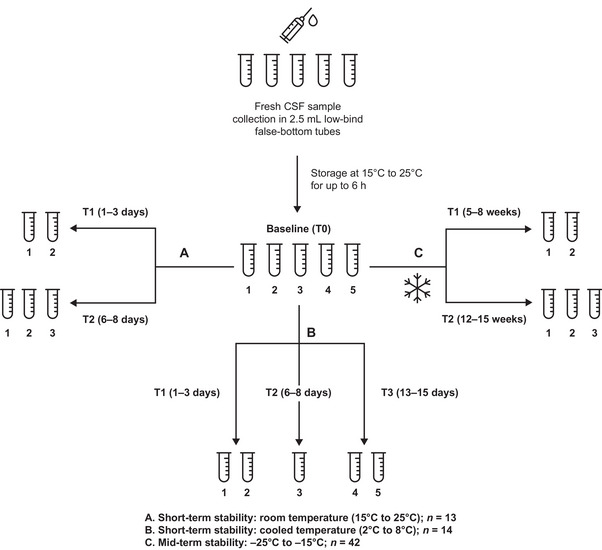
Schematic illustration of the workflow of the sample stability experiments. Aliquots 1–5 represent five samples of fresh CSF collected from each study subject directly in 2.5‐mL low‐bind false‐bottom tubes. Numbers are given randomly and are not related to the collection order. According to the study design, each aliquot was measured twice, once at baseline and once at one of the follow‐up studies points T1–T3. Aβ42, amyloid beta 1–42; CSF, cerebrospinal fluid; p‐tau181, tau phosphorylated at a threonine residue at position 181; T, timepoint; t‐tau, total tau.

For each subject, five aliquots (2.5 mL) of fresh CSF samples were collected by lumbar puncture using a gravity drip protocol. After discarding the first 2 mL, the remainder was dispensed directly into five 2.5‐mL, low‐bind, false‐bottom tubes (Sarstedt, #63.614.699), following immunoassay manufacturer protocols. Samples were stored at room temperature (15–25°C) without further handling (no centrifugation, mixing, or tube transfers) for up to 6 h before baseline biomarker measurement (timepoint [T] 0). All aliquots were transferred to their assigned storage conditions within 2 h after T0. Follow‐up measurements were performed as outlined below and in Figure 1.

For the room temperature experiment (15–25°C), sample aliquots were incubated at 25°C; aliquots 1–2 were measured at T1 (1–3 days after T0) and aliquots 3–5 were measured at T2 (6–8 days after T0). For the cooled temperature (2–8°C) and mid‐term stability (−25°C to −15°C) experiments, samples were stored at the respective temperatures. In the cooled temperature (2–8°C) experiment, aliquots 1–2 were measured at T1 (1–3 days after T0), aliquot 3 at T2 (6–8 days after T0), and aliquots 4–5 at T3 (13–15 days after T0). In the mid‐term stability (−25°C to −15°C) experiment, aliquots 1–2 were measured at T1 (5–8 weeks after T0) and aliquots 3–5 at T2 (12–15 weeks after T0). Before measurement, frozen samples were thawed at 20–25°C for 30 min in an upright position, followed by mixing on a roller mixer for 20 min.

In all experiments, temperatures were maintained within predefined ranges and were monitored continuously. The concentrations of the Aβ42, p‐tau181, and t‐tau biomarkers were measured using the Elecsys β‐Amyloid (1–42) CSF II, Elecsys Phospho‐Tau (181P) CSF, and Elecsys Total‐Tau CSF immunoassays, respectively. Storage durations varied between subjects due to prospective enrollment and synchronized measurement runs; thus, time points were not equidistant, allowing broader temporal coverage.

Due to the limited number of aliquots per subject, each CSF sample was measured twice: once at baseline and at one designated follow‐up time point. This design was supported by prior feasibility studies, which showed no effect of repeated measurement on samples prepared according to the described procedure with a fill volume of 2.5 mL. In addition, as the study was prospective, measurements took place over several months. Daily quality control (QC) assessments were performed using two control samples, with laboratory‐specific acceptable ranges defined according to the Clinical and Laboratory Standards Institute (CLSI) C24‐Ed4 guideline and package insert recommendations. Samples were analyzed only when QC results were within these specified ranges. Any QC violations prompted immediate corrective actions, such as re‐calibration, before proceeding with sample analysis.

### 2.3 Statistical analysis

The statistical analyses performed in this study were pre‐specified in consultation with regulatory agencies. Systematic effect of sample aging within ±10% was considered acceptable.

#### 2.3.1 Short‐term stability

The main objective of the short‐term stability experiments was to estimate storage effect in terms of changes in percent concentration recoveries and estimation of storage duration based on the results. Short‐term stability was evaluated using ordinary least squares (OLS) linear regression, in accordance with regulatory recommendations and the (now superseded) CLSI EP25‐A3 guideline.

For each subject and time point (T1–T3), recovery was calculated as the mean analyte concentration at each time point divided by the baseline (T0) mean concentration, expressed as a percentage: Recovery at T_x_ = C[T_x_] / C[T_0_]*100%, where C[T_x_] is the mean concentration at time point T_x_ (*x *= 1, 2, 3). Storage duration was determined as the time difference (in days) between the baseline and follow‐up measurements for each subject and time point.

For each storage condition, all subject data were pooled, and an OLS regression model was fitted: Recovery = β_0_ + β_1_*t, where t indicates storage duration. Following CLSI EP25‐A3 recommendations, and if the regression slope was statistically significant (*p* < 0.05), conservative estimate of maximum allowable storage duration was defined as the intersection of the acceptance criterion (1.00 ± 0.10) with the 95% one‐sided mean confidence interval (CI) of the regression line. If the slope was not significant, the analyte was considered stable over the tested storage period.

Although OLS regression does not account for within‐subject correlation of repeated measures, the point estimates of regression coefficients remain unbiased if the model is correctly specified. However, standard errors may be underestimated, leading to potentially anti‐conservative inference. To address this, sensitivity analyses were performed, and CIs were additionally derived using a bootstrap approach to account for within‐subject correlation.

#### 2.3.2 Mid‐term stability

For the mid‐term stability experiment, which evaluated the combined effects of storage at −25°C to −15°C and a single freeze/thaw cycle, a method comparison approach was used for the primary analysis, as recommended in CLSI EP09c. Given the anticipated impact of freezing on the values and variability of Aβ42 measurements, a larger cohort was enrolled, and an alternative statistical approach was adopted to allow for a more powerful statistical analysis and enable a more precise estimation of the freeze/thaw effect.

To assess stability after storage for 5–8 weeks (T1) and one freeze/thaw cycle, measurements at T1 were compared with baseline values at T0. Similarly, stability after storage for 12–15 weeks (T2) was evaluated by comparing concentrations at T2 with those at T0. For each subject, the mean concentration at T0 (X) was paired with the corresponding mean concentration at T1 or T2 (Y), and Passing–Bablok regression was performed (Y = a + bX) to account for measurement errors in variables X and Y.

The proportional bias at a pre‐defined concentration (M; Table S1) was calculated using the following formula: bias (%) = 100% (a + [b – 1] × M) / M. Acceptance criteria were defined as where the slope estimate was within 1.00 ± 0.07 and the estimate of the proportional bias was within ±7%. The narrow acceptance criteria ensured high probability (>80%) to detect an unacceptable systematic storage effect of ±10%. Multiplicity adjustment was not applied, as classical significance testing was not conducted. Instead, point estimates were compared directly to predefined acceptance criteria; the study was considered unsuccessful if these criteria were not met.

In addition to the primary analyses, concentration recoveries at each study time point were summarized using descriptive tables and boxplots.

## 3 RESULTS

### 3.1 Sample timing and immunoassay performance

The median (interquartile range) time interval between sample collection and baseline measurement was 3 h (2–4 h). For three of 42 subjects, this time interval exceeded 6 h; for two of these subjects, the interval was between 7 and 8 h.

Throughout the study, the Elecsys CSF immunoassays exhibited high precision and consistency. The highest variability among the five baseline measurements per subject was observed in the mid‐term stability experiment. Across 42 subjects, the median (min, max) coefficient of variation was 1.39% (0.37, 8.30) for Aβ42, 1.08% (0.41, 2.55) for p‐tau181, and 1.10% (0.26, 2.55) for t‐tau. The strong correlation observed between baseline and follow‐up measurements over several months in the mid‐term sample stability study indicated the low day‐to‐day variability of the immunoassays.

### 3.2 Short‐term stability

#### 3.2.1 Baseline demographics and CSF biomarker data

A total of 27 subjects were enrolled in the short‐term stability experiments (13 in the room temperature experiment [15–25°C] and 14 in the cooled temperature experiment [2–8°C]) with a mean age (standard deviation [SD]) of 66.6 (11.3) and 67.8 (8.07) years and a mean MMSE score (SD) of 26.2 (2.89) and 26.0 (2.51) in the room and cooled temperature experiments, respectively. The proportion of females was 30.8% in the room temperature experiment and 64.3% in the cooled temperature experiment.

In the room temperature experiment, one and two missing concentrations were observed for Aβ42 at the follow‐up study points T1 and T2, respectively. In the cooled temperature experiment, one missing concentration was observed for p‐tau181 at the follow‐up study point T1, and one for Aβ42 at the follow‐up study point T2. Missing data were due to protocol deviations that led to invalid results. There were no missing data for t‐tau at baseline or follow‐up in the room and cooled temperature experiments.

The demographics and baseline CSF biomarker data of all enrolled subjects are summarized in Table S2 and Table 1, respectively.

**TABLE 1:** Baseline CSF biomarker data for the short‐ and mid‐term stability experiments.

	Short‐term stability		Mid‐term stability
Median biomarker concentration	Room temperature (15–25°C)	Cooled temperature (2–8°C)	−25°C to −15°C
n*	13	14	42
Aβ42, pg/mL (range)	890 (404–1609)	693 (540–1932)	837 (333–2411)
p‐tau181, pg/mL (range)	18.8 (8.1–42.0)	21.3 (10.3–38.6)	23.6 (8.3–93.9)
t‐tau, pg/mL (range)	211 (96.2–395.0)	245 (131.0–373.0)	275 (108.0–933.0)

*Note*: *n denotes the number of subjects with available valid baseline measurements for at least one biomarker.

Abbreviations: Aβ42, amyloid beta 1**–**42; CSF, cerebrospinal fluid; p‐tau181, tau phosphorylated at a threonine residue at position 181; t‐tau, total tau.

#### 3.2.2 Room temperature experiment (15–25°C)

**FIGURE 2:**
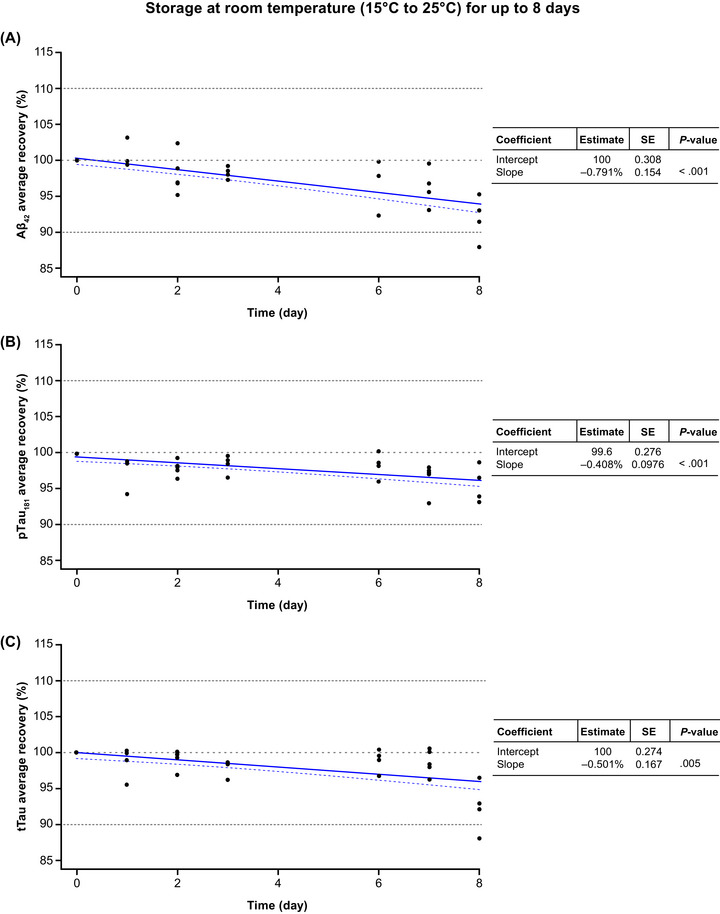
Short‐term stability of (A) Aβ42, (B) p‐tau181, and (C) t‐tau in CSF samples stored at room temperature (15–25°C) for up to 8 days. Aβ42, amyloid beta 1–42; CSF, cerebrospinal fluid; p‐tau181, tau phosphorylated at a threonine residue at position 181; SE, standard error; t‐tau, total tau.

Storage effects (slopes interpreted as estimated change in percentage recovery per day) observed at 15–25°C were significantly different from zero for all three biomarkers (Figure 2). The effect sizes observed for p‐tau181 and t‐tau were small, whereas a slightly larger storage effect was observed for Aβ42 (slope estimate: –0.791%; *p* < 0.001) compared with p‐tau181 (slope estimate: −0.408%; *p* < 0.001) and t‐tau (slope estimate: –0.501%; *p* = 0.005). The estimated storage duration (where the crossing point of the confidence bound with horizontal line recovery equals 90%) was above the maximum tested storage time (*T*_max_ = 8 days) and, therefore, all three biomarkers were considered stable at 15–25°C for up to 8 days. The median (min, max) average concentration recoveries at the respective follow‐up study points T1 and T2 were: 98.7% (95.2, 93.0) and 95.3% (87.9, 99.8) for Aβ42; 98.4% (94.4, 99.5) and 97.4% (93.1, 100.0) for p‐tau181; and 98.9% (95.5, 100.0) and 98.0% (88.1, 101.0) for t‐tau (Table S3). For Aβ42, the median concentration recovery showed slight but systematic decline after Day 6 of storage (Figure S1). Clear concentration drift and one concentration recovery below 90% were observed at Day 8 for Aβ42 and t‐tau (Figure 2).

#### 3.2.3 Cooled temperature experiment (2–8°C)

**FIGURE 3:**
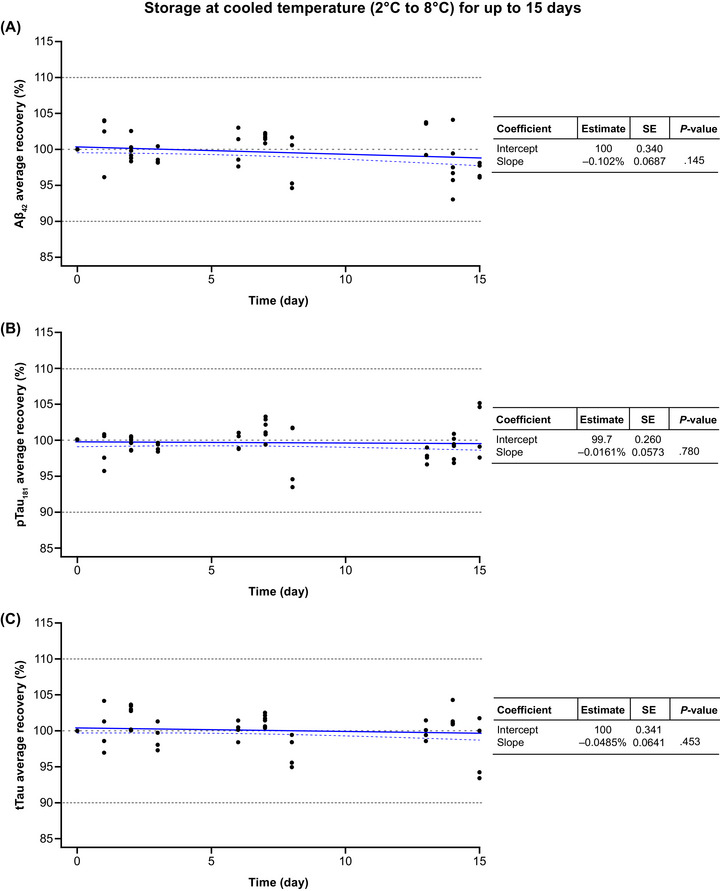
Short‐term stability of (A) Aβ42, (B) p‐tau181, and (C) t‐tau in CSF samples stored at cooled temperature (2–8°C) for up to 15 days. Aβ42, amyloid beta 1–42; CSF, cerebrospinal fluid; p‐tau181, tau phosphorylated at a threonine residue at position 181; SE, standard error; t‐tau, total tau.

All three biomarkers were stable when stored at 2–8°C for up to 15 days (Figure 3). The observed storage effects were not significantly different from zero for all three biomarkers: Aβ42 (slope estimate: −0.102%; *p* = 0.145), p‐tau181 (slope estimate: −0.0161%; *p* = 0.780), and t‐tau (slope estimate: –0.0485%; *p* = 0.453). The median (min, max) average concentration recoveries at the respective follow‐up study points T1, T2, and T3 were: 99.5% (96.1, 104.0), 101.0% (94.5, 103.0), and 97.9% (93.0, 104.0) for Aβ42; 99.4% (95.6, 101.0), 101.0% (93.4, 103.0), and 98.9% (96.5, 105.0) for p‐tau181; and 100.0% (94.9, 103.0), 100.0% (94.9, 103.0), and 101.0% (93.4, 104.0) for t‐tau (Table S3). All concentration recoveries were within 100 ± 10% (Figure S1).

### 3.3 Mid‐term stability

#### 3.3.1 Baseline demographics and CSF biomarker data

A total of 42 subjects were enrolled in the mid‐term stability experiments with a mean age (SD) of 67.5 (8.58) years and a mean MMSE score (SD) of 23.0 (5.53); 35.7% of the enrolled subjects were female. At baseline, one missing concentration was observed for Aβ42, whereas Aβ42 concentrations were available for only 38 and 33 subjects at the follow‐up study points T1 and T2, respectively. Missing data were due to protocol deviations that led to invalid results. There were no missing data for p‐tau181 and t‐tau at baseline or follow‐up. The demographics and CSF biomarker data of all enrolled subjects are summarized in Table S2 and Table 1, respectively.

#### 3.3.2 Storage at −25°C to −15°C for up to 8 weeks and one freeze/thaw cycle (T0 vs T1)

**FIGURE 4:**
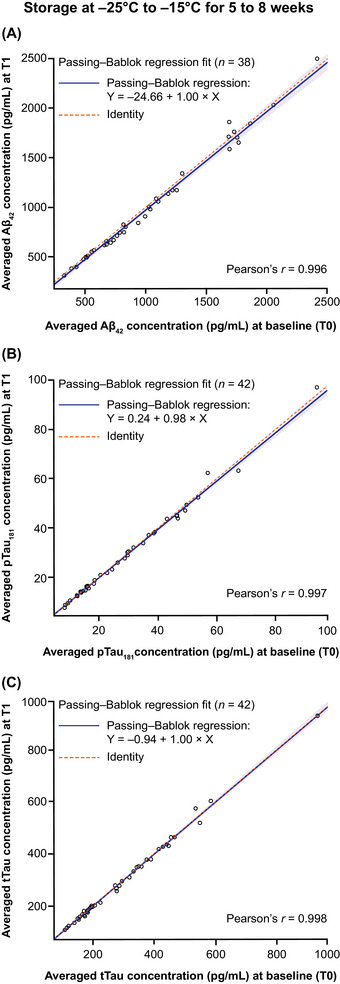
Mid‐term stability of (A) Aβ42, (B) p‐tau181, and (C) t‐tau in CSF samples stored at −25°C to −15°C for 5–8 weeks (comparison T0 vs T1). X indicates the averaged concentrations at T0, and Y indicates the averaged concentrations at T1. Aβ42, amyloid beta 1–42; CSF, cerebrospinal fluid; p‐tau181, tau phosphorylated at a threonine residue at position 181; T, timepoint; t‐tau, total tau.

All samples were stable after storage at −25°C to −15°C for up to 8 weeks and one freeze/thaw cycle (Figure 4). The concentrations observed at T0 and T1 were highly correlated (Pearson's *r* > 0.99 for all biomarkers) and slope estimates were close to 1.000 (Aβ_42_: 0.996; p‐tau181: 0.979; t‐tau: 1.000). The largest bias estimate at the pre‐specified concentration was observed for Aβ42 (bias: −2.840%; 95% CI −5.000, −0.389) at a concentration of 1030 pg/mL (Table S4). The median (min, max) average concentration recovery at T1 was 96.0% (89.7, 110.0) (Table S3); of the 38 concentration recoveries, 37 were within 100 ± 10% (Figure S1). For p‐tau181 and t‐tau, the observed bias estimates were not significantly different from 0% (p‐tau181, bias: −1.190%; 95% CI –2.530, 0.950 at 27 pg/mL; t‐tau, bias: −0.336%; 95% CI −1.680, 0.694 at 300 pg/mL) (Table S4). The median (min, max) average concentration recoveries at T1 were 99.1% (91.6, 109.0) and 99.7% (92.0, 107.0) for p‐tau181 and t‐tau, respectively (Table S3, Figure S1).

#### 3.3.3 Storage at −25°C to −15°C for 12–15 weeks and one freeze/thaw cycle (T0 vs T2)

**FIGURE 5:**
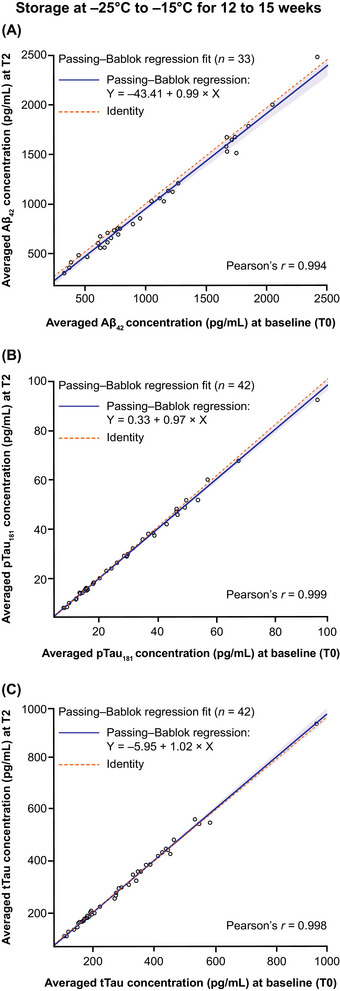
Mid‐term stability of (A) Aβ42, (B) p‐tau181, and (C) t‐tau in CSF samples stored at −25°C to −15°C for 12–15 weeks (comparison T0 vs T2). X indicates the averaged concentrations at T0, and Y indicates the averaged concentrations at T2. Aβ_42_, amyloid beta 1–42; CSF, cerebrospinal fluid; p‐tau181, tau phosphorylated at a threonine residue at position 181; T, timepoint; t‐tau, total tau.

The observed effect of freezing and storage at −25°C to −15°C for 12–15 weeks was negligible for p‐tau181 and t‐tau, whereas a small but significant systematic decline was observed for Aβ42 under the same conditions (Figure 5). The concentrations observed at T0 and T2 were highly correlated (Pearson's *r* > 0.99 for all biomarkers) and slope estimates were close to 1.000 (Aβ42: 0.992; p‐tau181: 0.971; t‐tau: 1.020). The largest bias estimate at the pre‐specified concentration was observed for Aβ42 (bias: −5.000%; 95% CI −7.830, –2.830 at 1030 pg/mL) (Table S5). For Aβ42, the median (min, max) average concentration recovery was 95.7% (84.0, 105.0) (Table S3), and ≈30% of Aβ42 concentration recoveries were below 90% (Figure S1). For p‐tau181 and t‐tau, the median (min, max) average concentration recoveries were 98.9% (93.9, 105.0) and 99.6% (92.8, 106.0), respectively (Table S3).

## 4 DISCUSSION

This prospective study explored the stability of Aβ42, p‐tau181, and t‐tau in fresh CSF samples collected from subjects being evaluated for AD. The findings indicated that Aβ42 was less stable than p‐tau181 and t‐tau after storage at room temperature (15–25°C) for up to 8 days, with a systematic decline in the average concentration recovery after Day 6 of storage. The cooled temperature range of 2–8°C was found to be the optimal storage condition for fresh CSF, as all biomarkers were stable for up to 15 days. In the mid‐term stability experiment, the concentrations of all three biomarkers were not affected after storage at −25°C to −15°C for up to 8 weeks and one freeze/thaw cycle. Storage at the same temperature for 12–15 weeks had a negligible effect on the concentrations of p‐tau181 and t‐tau, whereas the concentration of Aβ42 was affected more, with an approximate systematic decline of 5% from baseline (T0) and a concentration reduction of ≥10% in some individual samples. Therefore, as a conservative approach, it is recommended to store fresh CSF samples for up to 5 days at room temperature (15–25°C), for up to 15 days at cooled temperature (2–8°C), and for up to 8 weeks at −25°C to −15°C.

Sample stability and the effect of pre‐analytical variables on CSF biomarker measurements for AD have been explored in several studies, sometimes with inconsistent results.^26^, ^34^, ^35^, ^36^, ^37^ Most published studies rely on samples that experienced at least one freeze/thaw cycle before baseline measurement,^26^ primarily due to the challenges in obtaining sufficient volumes of fresh CSF directly from patients. Most importantly, both sample stability and freeze/thaw effects are highly dependent on factors such as tube type, fill volume, and specific pre‐analytical procedures.^26^

As the routine‐use procedure recommended in the Elecsys CSF immunoassay package inserts enables collection, transport, and measurement of fresh, never‐frozen CSF samples, a dedicated stability study was needed to explore analyte stability under these conditions. The present study was conducted in subjects representative of the intended‐use population, that is, subjects being evaluated for AD, ensuring the clinical relevance of the cohort. All samples were processed in accordance with the protocols specified in the Elecsys CSF immunoassay package inserts, and the combination of robust study design and high analytical performance supported clear interpretation of the results.

Aβ42 was the biomarker most affected by pre‐analytical factors, which is consistent with the previous literature.^29^, ^30^, ^33^, ^38^ A study conducted to evaluate the sample stability of CSF Aβ42, p‐tau181, and t‐tau using the Lumipulse G600II immunoassay (Fujirebio Diagnostics, Inc.) found that the temperature and duration of storage significantly influenced Aβ42, whereas p‐tau181 and t‐tau were not affected by any of the tested storage conditions.^38^ As Aβ42 has a strong tendency to form aggregates,^29^ lower stability results are often observed compared with tau biomarkers. Nevertheless, the results observed for Aβ42 in this analysis were good overall, and the small freeze/thaw effect could be attributed to the low‐bind surface of the tubes used according to the manufacturer‐recommended routine‐use pre‐analytical protocol and the optimal fill volume recommended in the package insert of the Elecsys β‐Amyloid (1–42) CSF II immunoassay. In addition, the observed storage effects were small enough so that the cutoff values established for the fresh CSF samples could be applied for samples that have been frozen. Previous research has shown that Aβ42 concentration varied depending on the tube type used; for example, variation was reduced when using a low‐bind, false‐bottom tube.^33^ It was also found that filling the tubes to maximum levels before storing at 2–8°C resolved the variation in Aβ42 levels.^33^ Therefore, a standardized pre‐analytical protocol is essential to ensure accuracy when measuring AD biomarkers, particularly Aβ42.

Small biases from sample storage could shift biomarker measurements, potentially affecting classification for cases near the clinical cutoff and ultimately diagnostic decision‐making. The risk of re‐classification depends on the density of observations around this threshold. A previous study using computer simulations to assess how analytical and pre‐analytical variability, including storage effects, influence the performance of three AD biomarkers (CSF p‐tau/Aβ42, CSF Aβ42/40, and plasma Aβ42/40) showed that the CSF p‐tau/Aβ42 ratio is the most robust among these biomarkers, showing minimal susceptibility to such biases.^39^ In the present study, pre‐specified criteria were applied, allowing no more than ±10% deviation in biomarker values due to storage effects while recognizing that these biomarkers are usually interpreted as ratios. According to Rabe et al., even when tau and Aβ42 biomarkers are affected in opposite directions, resulting in a combined bias of around 22% in the ratio, the diagnostic performance of the CSF p‐tau/Aβ42 or t‐tau/Aβ42 ratio remains considerably stable.^39^ Changes in positive and negative agreement with amyloid PET do not exceed 10% under these conditions. Collectively, these findings provide strong evidence for the robustness of the CSF p‐tau/Aβ42 and t‐tau/Aβ42 ratios as diagnostic biomarkers. This supports its continued use in clinical and laboratory settings, reassuring clinicians and laboratories of its reliability, even in the presence of minor analytical or pre‐analytical variation.

This study also demonstrated the high performance of the Elecsys CSF immunoassays in measuring biomarker levels in fresh CSF samples stored under different conditions and provides clear instructions on routine pre‐analytical sample handling and storage conditions to ensure the diagnostic accuracy of the immunoassays. These findings suggest that the Elecsys immunoassays along with the manufacturer‐recommended pre‐analytical protocol can be used in clinical practice to determine AD biomarker levels with high accuracy, serving as an important asset in the AD diagnostic pathway. In general, stability studies can be confounded by the inherent variability of the immunoassay, which may affect the accuracy and interpretation of the results^37^; however, the Elecsys CSF immunoassays have been shown to offer high precision and good lot‐to‐lot comparability with low day‐to‐day variability, indicating that the results of this study can be ascribed to the effects of storage conditions.^24^

Furthermore, at the time this study was conducted, no CLSI guideline was available to specifically address sample stability evaluation studies. The statistical methods applied here were developed in consultation with regulatory authorities and followed approaches similar to those outlined in the CLSI EP25 (reagent stability) and CLSI EP09 (method comparison) guidelines. Both the regression‐based analyses and descriptive summaries of concentration recoveries yielded consistent results, underscoring the robustness of the findings.

### 4.1 Limitations

This prospective sample stability study employed the recommended routine‐use pre‐analytical protocol for sample collection in the clinical intended‐use population (subjects being evaluated for AD). Subjects beyond the intended‐use population were considered only in cases where obtaining desired biomarker level ranges was challenging; however, no subject outside the clinical intended‐use population was recruited.

Although the sample size in this study is typical (*n* = 12–14 for short‐term stability) or relatively large (*n* = 42 for mid‐term stability) compared with most sample stability studies, it is still insufficient to reliably assess the potential influence of factors such as age, gender, comorbidities, or cognitive status on sample stability. Moreover, as the primary focus of the study was to examine the pre‐analytical stability of CSF biomarkers, additional neuropsychologic assessments were not captured. Further research is needed to evaluate the impact of these variables, potentially considering a more comprehensive cognitive assessment of the enrolled subjects using additional neuropsychologic tests. To partially address this limitation, we examined storage effects across different concentration ranges (low, medium, and high) for each biomarker and found no evidence that concentration level influenced the results.

Samples in this study were handled according to the guidance in the Elecsys CSF immunoassay package inserts. Deviating from this recommended handling protocol may impact the analyte measurement, which could in turn affect sample stability and lead to different results, thereby reducing the diagnostic accuracy of the immunoassays. Thus, for optimal immunoassay performance, users are limited to use sterile low‐bind tubes for CSF sample collection and storage under the recommended conditions only. Moreover, the study did not investigate the long‐term stability or the stability of the three CSF biomarkers following storage at −80°C, which is common practice in clinical studies, as the routine‐use, low‐bind collection tube used in this study has not been validated by the manufacturer for use at such low temperatures. Therefore, the use of the low‐bind tube for CSF sample collection and storage is limited to facilities where storage at −20 ± 5°C is possible. Furthermore, sample storage under these conditions (−20 ± 5°C) should be limited to up to 8 weeks, as long‐term storage effects on sample stability remain to be explored; this restricts the use of the immunoassays and the pre‐analytical protocol to cases where samples can be tested within the indicated timeframe. Further studies will be needed to explore the effect of temperatures below −25°C as well as long‐term storage (>15 weeks) on the stability of Aβ42, p‐tau181, and t‐tau in fresh CSF samples handled according to the routine‐use pre‐analytical protocol.

In conclusion, all three biomarkers demonstrated sufficient stability at each storage condition tested. As a conservative approach, it is recommended that CSF samples are stored at room temperature (15–25°C) for up to 5 days, at cooled temperature (2–8°C) for up to 15 days, and at −25°C to −15°C for up to 8 weeks. Taken together, these results provide important information regarding the optimal pre‐analytical handling and storage conditions of CSF samples. These findings are critical for ensuring the best performance of the Elecsys CSF immunoassays in order to enable diagnostic accuracy of detecting amyloid positivity in subjects being evaluated for AD.

## CONFLICT OF INTEREST STATEMENT

E.M., S.M., and S.R. are full‐time employees of Roche Diagnostics GmbH (Penzberg, Germany) and hold shares in F. Hoffmann‐La Roche. I.S. is a full‐time employee of Roche Diagnostics International Ltd (Rotkreuz, Switzerland). O.G. and P.S. have no conflicts of interest. T.G. has received grants to his institution from Biogen, Eisai, Eli Lilly, and Roche Diagnostics. Outside the submitted work, T.G. received consulting fees from Acumen, Advantage Therapeutics, Alector, Anavex, Biogen, BMS, Cogthera, Eisai, Eli Lilly, Functional Neuromodulation, Grifols, Janssen, Neurimmune, Noselab, Novo Nordisk, Roche Diagnostics, and Roche Pharma; lecture fees from Anavex, Cogthera, Eisai, Eli Lilly, FEO, Grifols, Pfizer, Roche Pharma, Schwabe, and Synlab; and has received grants to his institution from Biogen, Eisai, and Eli Lilly. Author disclosures are available in the Supporting Information.

## ETHICS APPROVAL AND CONSENT TO PARTICIPATE

All samples used were native samples that were collected prospectively and donated to the biobank of the study site. Informed consent from each subject, allowing the use of sample material for research, was required prior to the study. All samples and required clinical information were pseudonymized. The study was approved by the ethics committee of the Technical University of Munich (vote no. 637/20).

## Supporting information



Supporting information

Supporting information
