# Supplementary material for: Elecsys CSF AD immunoassays: Sample stability for a new pre‐analytical protocol for fresh CSF
Source: Alzheimers Dement. 2025 Oct 17;21(10):e70797. doi: 10.1002/alz.70797 (PMC12531901; doi:10.1002/alz.70797)

**Supplementary materials**

# Supplementary Methods

## Statistical approach

### Short-term stability

An additional approach used to describe recovery in the short-term stability experiments comprised calculation of the mean, standard deviation, median, minimum, and maximum.

### Mid-term stability

For mid-term stability experiments, two further approaches were used to describe recovery. The second approach comprised calculation of concentration recoveries at the follow-up study point and description of their distributions (mean, standard deviation, median, minimum, and maximum), while the third approach fitted recovery (Y) ~ storage duration (X) to the data to investigate the dependency between storage duration and the concentration recovery linear regression model. This analysis was not pre-specified in the statistical analysis plan and was for descriptive purposes only.

**Supplemental Table 1:** Pre-defined concentrations of Aβ_42_, pTau_181_, and tTau for the calculation of proportional bias

| **Biomarker** | **M (pg/mL)** |
| --- | --- |
| Aβ_42_ | 1030 |
| pTau_181_ | 27 |
| tTau | 300 |

Abbreviations: Aβ_42_, β-amyloid(1–42); M, pre-defined concentration; pTau_181_, tau phosphorylated at a threonine residue at position 181; tTau, total tau

**Supplemental Table 2:** Demographic characteristics of subjects enrolled in the short- and mid-term stability experiments

|  | **Short-term stability** | | **Mid-term stability** |
| --- | --- | --- | --- |
|  | **Room temperature  (15°C to 25°C)** | **Cooled temperature  (2°C to 8°C)** | **–25°C to –15°C** |
| **Total/missing, *n*** | **13/0** | **14/0** | **42/0** |
| **Age, years** |  |  |  |
| Mean (SD) | 66.6 (11.30) | 67.8 (8.10) | 67.5 (8.58) |
| Median | 62.0 | 65.0 | 67.0 |
| Min | 51.0 | 57.0 | 53.0 |
| Max | 86.0 | 79.0 | 83.0 |
| **Sex, *n* (%)** | | | |
| Female | 4 (30.8) | 9 (64.3) | 15 (35.7) |
| Male | 9 (69.2) | 5 (35.7) | 27 (64.3) |
| **Race, *n* (%)** | | | |
| White | 13 (100.0) | 14 (100.0) | 41 (97.6) |
| White, other | 0 | 0 | 1 (2.38) |
| **MMSE score** | | | |
| Mean (SD) | 26.2 (2.89) | 26.0 (2.51) | 23.0 (5.53) |
| Median | 26.0 | 26.0 | 24.0 |
| Min | 19.0 | 22.0 | 4.0 |
| Max | 30.0 | 30.0 | 29.0 |

Abbreviations: Max, maximum; min, minimum; MMSE, Mini-Mental State Examination; *n*, number of subjects; SD, standard deviation

**Supplemental Table 3:** Averaged recoveries of Aβ_42_, pTau_181_, and tTau at room temperature (15°C to 25°C), cooled temperature (2°C to 8°C), and –25°C to –15°C

|  | **T1** | **T2** | | **T3** |
| --- | --- | --- | --- | --- |
| **Averaged recoveries at room temperature  (15°C to 25°C)** | **1–3 days** | **6–8 days** | | **—** |
| **Aβ_42_** | | | | |
| Missing/non-missing, *n/N* | 1/12 | 2/11 | — | |
| Mean (SD), % | 98.8 (2.28) | 94.8 (3.64) | — | |
| Median, % | 98.7 | 95.3 | — | |
| Min−max, % | 95.2−103.0 | 87.9−99.8 | — | |
| **pTau_181_** | | | | |
| Missing/non-missing, *n/N* | 1/12 | 0/13 | — | |
| Mean (SD), % | 98.0 (1.50) | 96.9 (2.22) | — | |
| Median, % | 98.4 | 97.4 | — | |
| Min−max, % | 94.4–99.7 | 93.1–100.0 | — | |
| **tTau** | | | | |
| Missing/non-missing, *n/N* | 0/13 | 0/13 | — | |
| Mean (SD), % | 98.6 (1.52) | 96.8 (3.73) | — | |
| Median, % | 98.9 | 98.0 | — | |
| Min−max, % | 95.5–100.0 | 88.1–101.0 | — | |
| **Averaged recoveries at cooled temperature  (2°C to 8°C)** | **1–3 days** | **6–8 days** | | **13–15 days** |
| **Aβ_42_** | | | | |
| Missing/non-missing, *n/N* | 0/14 | 1/13 | 0/14 | |
| Mean (SD), % | 100.0 (2.37) | 100.0 (2.71) | 98.9 (3.54) | |
| Median, % | 99.5 | 101.0 | 97.9 | |
| Min−max, % | 96.1–104.0 | 94.6–103.0 | 93.0–104.0 | |
| **pTau_181_** | | | | |
| Missing/non-missing, *n/N* | 0/14 | 0/14 | 0/14 | |
| Mean (SD), % | 99.1 (1.38) | 99.9 (2.87) | 99.3 (2.64) | |
| Median, % | 99.4 | 101.0 | 98.9 | |
| Min−max, % | 95.6–101.0 | 93.4–103.0 | 96.5–105.0 | |
| **tTau** | | | | |
| Missing/non-missing, *n/N* | 0/14 | 0/14 | 0/14 | |
| Mean (SD), % | 101.0 (2.44) | 99.8 (2.30) | 99.9 (2.89) | |
| Median, % | 101.0 | 100.0 | 101.0 | |
| Min−max, % | 97.0–104.0 | 94.9–103.0 | 93.4–104.0 | |
| **Averaged recoveries at –25°C to –15°C** | **5–8 weeks** | **12–15 weeks** | | **—** |
| **Aβ_42_** | | | | |
| Missing/non-missing, *n/N* | 4/38 | 9/33 | — | |
| Mean (SD), % | 96.5 (4.26) | 94.5 (5.53) | — | |
| Median, % | 96.0 | 95.7 | — | |
| Min–max, % | 89.7–110.0 | 84.0–105.0 | — | |
| **pTau_181_** | | | | |
| Missing/non-missing, *n/N* | 0/42 | 0/42 | — | |
| Mean (SD), % | 99.2 (3.56) | 98.8 (2.86) | — | |
| Median, % | 99.1 | 98.9 | — | |
| Min–max, % | 91.6–109.0 | 93.9–105.0 | — | |
| **tTau** | | | | |
| Missing/non-missing, *n/N* | 0/42 | 0/42 | — | |
| Mean (SD), % | 99.1 (3.11) | 99.4 (3.42) | — | |
| Median, % | 99.7 | 99.6 | — | |
| Min–max, % | 92.0–107.0 | 92.8–106.0 | — | |

Abbreviations: Aβ_42_, β-amyloid(1–42); max, maximum; min, minimum; *N*, number of subjects; pTau_181_, tau phosphorylated at a threonine residue at position 181; SD, standard deviation; T, timepoint; tTau, total tau

**Supplemental Table 4:** Regression parameters and estimated percentage bias for Aβ_42_, pTau_181_, and tTau after freezing and storing at –25°C to –15°C for up to 8 weeks

|  | | **Estimate** | **95% CI*** | **Pearson** | **Spearman** | **Kendall** |
| --- | --- | --- | --- | --- | --- | --- |
| **Aβ_42_  (1030 pg/mL)** | Intercept | –24.700 | (–73.100, 3.680) | 0.996 | 0.991 | 0.948 |
|  | Slope | 0.996 | (0.953, 1.040) | — | — | — |
|  | Bias, % | –2.840 | (–5.000, –0.389) | — | — | — |
| **pTau_181_  (27 pg/mL)** | Intercept | 0.235 | (–0.200, 0.802) | 0.997 | 0.996 | 0.965 |
|  | Slope | 0.979 | (0.953, 1.010) | — | — | — |
|  | Bias, % | –1.190 | (–2.530, 0.950) | — | — | — |
| **tTau  (300 pg/mL)** | Intercept | –0.943 | (–8.200, 2.970) | 0.998 | 0.995 | 0.958 |
|  | Slope | 1.000 | (0.981, 1.030) | — | — | — |
|  | Bias, % | –0.336 | (–1.680, 0.694) | — | — | — |

*Note:* *95% CIs were calculated with the bootstrap method. Values in this table are rounded to three significant digits. Abbreviations: Aβ_42_, β-amyloid(1–42); CI, confidence interval; pTau_181_, tau phosphorylated at a threonine residue at position 181; tTau, total tau

**Supplemental Table 5:** Regression parameters and estimated percentage bias for Aβ_42_, pTau_181_, and tTau after freezing and storing at –25°C to –15°C for 12 to 15 weeks

|  | | **Estimate** | **95% CI*** | **Pearson** | **Spearman** | **Kendall** |
| --- | --- | --- | --- | --- | --- | --- |
| **Aβ_42_  (1030 pg/mL)** | Intercept | –43.4 | (–102.000, 17.200) | 0.994 | 0.982 | 0.909 |
|  | Slope | 0.992 | (0.929, 1.040) | — | — | — |
|  | Bias, % | –5.000 | (–7.830, –2.830) | — | — | — |
| **pTau_181_  (27 pg/mL)** | Intercept | 0.332 | (–0.210, 0.834) | 0.999 | 0.993 | 0.954 |
|  | Slope | 0.971 | (0.946, 1.000) | — | — | — |
|  | Bias, % | –1.620 | (–2.630, 0.050) | — | — | — |
| **tTau  (300 pg/mL)** | Intercept | –5.950 | (–12.000, 1.140) | 0.998 | 0.996 | 0.967 |
|  | Slope | 1.020 | (0.989, 1.050) | — | — | — |
|  | Bias, % | –0.148 | (–1.280, 1.240) | — | — | — |

*Note:* *95% CIs were calculated with the bootstrap method. Values in this table are rounded to three significant digits. Abbreviations: Aβ_42_, β-amyloid(1–42); CI, confidence interval; pTau_181_, tau phosphorylated at a threonine residue at position 181; tTau, total tau

**Supplemental Figure 1:** Concentration recoveries (%) for each biomarker observed at different follow-up study points. *N* denotes the number of subjects with available valid biomarker measurements at the follow-up study points. For Aβ_42_, one and two missing concentrations were observed at T1 and T2, respectively, at 15°C to 25°C (room temperature experiment); one missing concentration at T2 at 2°C to 8°C (cooled temperature experiment); four and nine missing concentrations at T1 and T2 at –25°C to –15°C (mid-term stability experiment), respectively*.* For pTau_181_, one missing concentration was observed at T1 at 2°C to 8°C (cooled temperature experiment). Missing data were due to protocol deviations that led to invalid results. There were no missing data for tTau at follow-up. Abbreviations: Aβ_42_, β-amyloid(1–42); *N*, number of subjects; pTau_181_, tau phosphorylated at a threonine residue at position 181; T, timepoint; tTau, total tau.


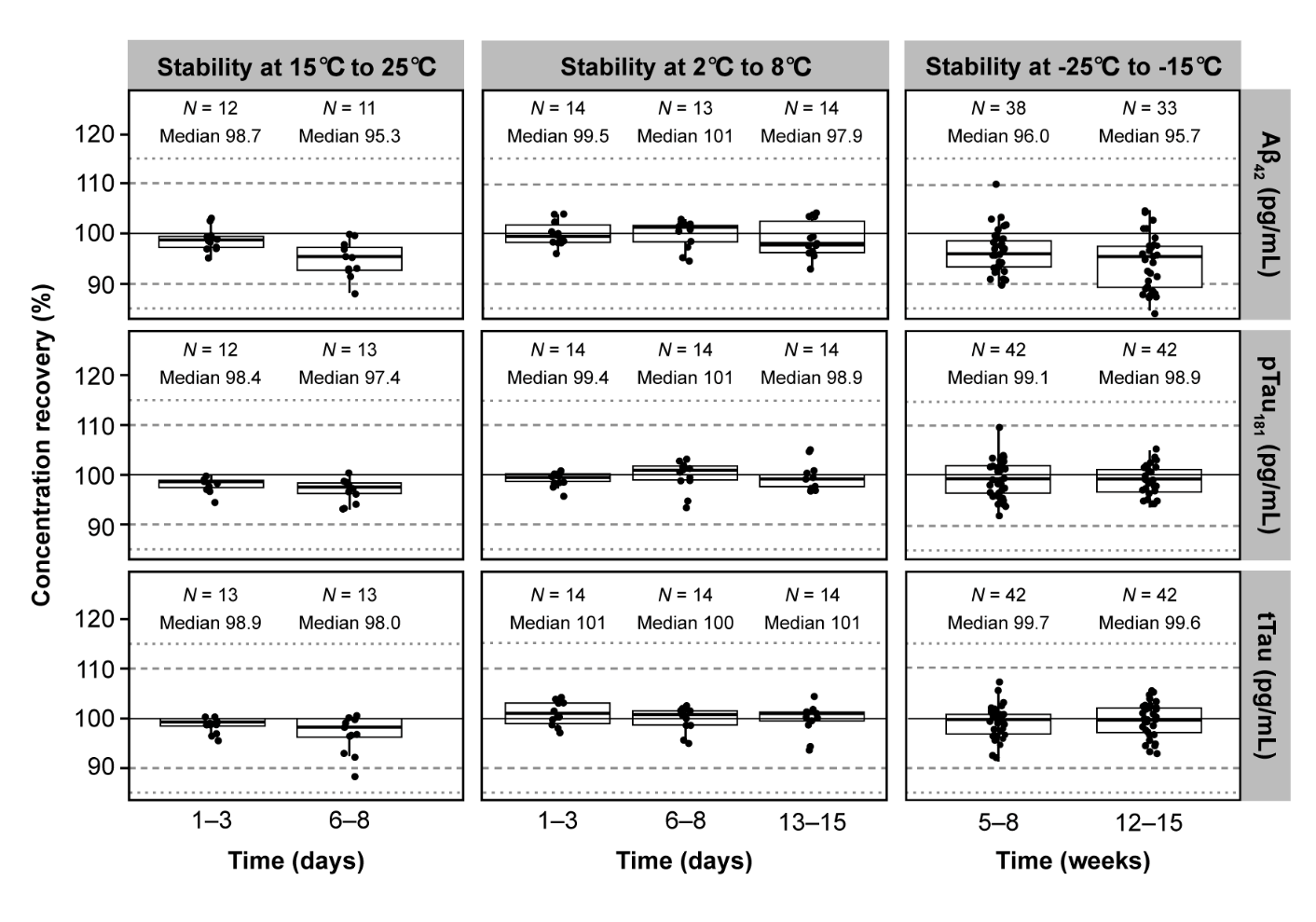

Supplement: Supplementary file 1 — Supporting information [file ALZ-21-e70797-s001.docx]
